# An Approach to Prevent Frailty in Community Dwelling Older Adults: a pilot study performed in Campania region in the framework of the PERSSILAA project

**Published:** 2019-01-06

**Authors:** M Cataldi, V De Luca, G Tramontano, C Del Giudice, I Grimaldi, P Cuccaro, P Speranza, G Iadicicco, V Iadicicco, F Carotenuto, PA Riccio, G Di Spigna, A Renzullo, L Vuolo, L Barrea, S Savastano, A Colao, G Liotta, G Iaccarino, P Abete, P Buono, M Vollenbroek-Hutten, M Illario

**Affiliations:** 1 Dipartimento di Neuroscienze, Scienze Riproduttive ed Odontostomatologiche, Università degli Studi di Napoli Federico II, Napoli, Italy; 2 Unità Operativa Semplice Ricerca e Sviluppo, Azienda Ospedaliera Universitaria Federico II, Napoli, Italy; 3 Unità Operativa Complessa Gestione Affari Generali, Azienda Ospedaliera Universitaria Federico II, Napoli, Italy; 4 Associazione Progetto Alfa ONLUS, Università degli Studi di Napoli Federico II, Napoli, Italy; 5 Dipartimento di Scienze Mediche Traslazionali, Università degli Studi di Napoli Federico II, Napoli, Italy; 6 Dipartimento di Endocrinologia ed Oncologia Molecolare e Clinica, Università degli Studi di Napoli Federico II, Napoli, Italy; 7 Dipartimento di Biomedicina e Prevenzione, Università di Roma, Tor Vergata, Roma, Italy; 8 Dipartimento di Scienze Biomediche Avanzate, Università degli Studi di Napoli Federico II, Napoli, Italy; 9 Dipartimento di Scienze Motorie e del Benessere, Università degli Studi di Napoli Parthenope, Napoli, Italy; 10 Institute for Biomedical Technology, University of Twente, Twente, The Netherlands; 11 Unità Operativa Dirigenziale Promozione e Potenziamento Programmi di Health Innovation, Regione Campania, Naples, Italy

**Keywords:** physical training, community, parish, ICT, physical function, physical performance

## Abstract

We developed and tested an innovative physical training method in older adults that embeds the gym program into everyday life in the most conservative way possible. Physical training was included in the activities of local parishes where older women from Southern Italy spend most of their free time and was delivered by trained physical therapists with the support of an ICT tool known as CoCo. 113 older women (aged 72.0 [69.0–75.0] years) noncompliant to conventional exercise programs participated to the study. 57 of them underwent the final anthropometric assessment and 50 the final physical tests. In study completers handgrip strength and physical performance evaluated with the chair-stand, the two minutes step and the chair-sit and -reach tests significantly improved. Quality of life as evaluated with the EuroQol-5dimension (EQ-5D) questionnaire improved as well. In conclusion, a training program designed to minimally impact on life habits of older people is effective in improving fitness in patients noncompliant to other to physical exercise programs.

## I. INTRODUCTION

A progressive decrease in physical function -the ability to carry out physical action from simple to very complex tasks [1]-takes place with ageing and is mainly due to inadequate physical activity and related sarcopenia (ICD-10 code-M62.84) [2]. Ageing-related physical activity decline increases frailty and disability, contributes to falls, and is associated with higher than normal cardiovascular risk [3]. Sarcopenic older adults with limited mobility have a shorter life expectancy than people of similar age but with a better preserved muscle mass [4]. The pathogenetic mechanism of age-related sarcopenia is still uncertain although a definite role can be ascribed to the progressive loss of fast-type motor neurons and of the fast myofibers with a compensatory increase of slow motorneurons and slow myofibers [5,6], to the negativization of protein synthesis balance in muscles [7,8], and to alterations in muscle mitochondria [9]. Because of the incomplete understanding of its pathophysiology, a rationale treatment of sarcopenia is still lacking. Nevertheless, it is largely acknowledged that this syndrome should be prevented before its occurrence with appropriate physical exercise programs and concurrent nutritional interventions [10,11]. The rationale behind the effectiveness of physical exercise is that like younger muscles, although probably with different molecular mechanisms, the neuromuscular unit may adapt to the increased functional demand by increasing muscle mass and strength [12]. The efficacy of a structured physical exercise intervention on muscle mass and strength in older people has been documented in randomized clinical trials [13, 14]. Importantly, physical exercise has been shown to improve survival [15] and to decrease the occurrence of falls [16–20]. However, the implementation of such a kind of interventions in clinical practice is often problematic because of a number of barriers that dramatically lower patient adherence [21–24].

Some of these barriers are infrastructural because in modern, not elder-friendly towns it can be difficult and expensive to go to the gym and sometimes the car may be needed [23]. Other barriers are directly related to ageing such as the lack of motivation due to social isolation and depression, and cognitive and memory difficulties. Several strategies have been suggested to improve adherence to the treatment [25,26]. Many of them include the use of Information and Communications Technology (ICT) supports with the aim of guiding patients in the execution of physical exercises and of increasing motivation by making the training session also a gaming activity as in *exergaming* [27–30]. However, the low computer literacy of older adults may represent an additional barrier to the implementation of ICT solutions.

Starting from the working hypothesis that the best way to maximize older patient cooperation to a health program was that this program does not substantially modify patient life habits we designed an intervention aiming to embed physical training sessions in the ordinary social activities of a group of older women in Italy. To this aim we identified the local parishes as a preferential location for our intervention because in Southern Italy older adults, especially women, gather there for a number of social and religious activities (e.g. playing cards, charity, etc.). Therefore, with the help of the parsons, we performed a pilot study to evaluate the effect of adding ICT-assisted gym sessions to the schedule of these social activities. To enhance the informatics literacy of the participants we also included in the program computer classes. This intervention was delivered as part of the activities of PERSSILAA (Personalised ICT Supported Service for Independent Living and Active Ageing) (https://perssilaa.com/) [31], a project of the A3 action group of the European Innovation Partnership on Active and Healthy Ageing (EIPonAHA) (https://ec.europa.eu/eip/ageing/home_en) [32].

## II. METHODOLOGY

### Study design

The present investigation was designed as an open, prospective, interventional pilot study. The study was carried out in 3 parishes (Confalone, Rogazionisti and Pilar) of the big Naples metropolitan area from January 2015 to June 2016. The study protocol n. 178/2014 was approved by the “Università Federico II” ethics committee and all the participants gave their informed consent to the experimentation. Procedures were in accordance with the 1964 Helsinki declaration and its later amendments or comparable ethical standards.

### Inclusion and Exclusion Criteria

Participants were recruited among the older adults attending their parish.

Inclusion criteria were: females, aged higher or equal to 65 years with an apparent good health status that had been non-compliant to physical exercise programs prescribed by their physicians.

Exclusion criteria were: one or more major cardiovascular events (including stroke and myocardial infarction) during the last 6 months, neoplastic or autoimmune diseases, and severe cognitive impairment including Alzheimer disease and other forms of dementia and invalidating neurological disorders including Parkinson’s disease. Bedridden patients and patients with major disability and mobility disorders were excluded as well.

### Study protocol

Volunteers were appointed for a first visit during which we performed a comprehensive evaluation of their medical, cognitive and fitness status by using health questionnaires and physical and instrumental examination as detailed in “*Variables*”. After completing the basal visit, subjects matching the inclusion and exclusion criteria, signed the informed consent and were enrolled to the the intervention described in the next paragraph. At the end of the treatment study participants were reevaluated in a final visit with the same protocol.

### Study Intervention

Study intervention consisted of exercise training and computer classes given twice a week at the local parish. During the exercise classes with the help of a licensed physical therapist study participants performed physical exercises based on the OTAGO exercise program [18,19]. This program consists of a set of leg muscle strengthening and balance retraining exercises progressing in difficulty, and a walking plan [33]. The *strength exercises* comprise calf rise, toe rise, front knee, back knee and side hip strengthening exercises. The *balance exercises* include Knee Bends, Stand To Sit backwards-, sideways-, stair-, heel- and toe- walking, walking and turning around, Heel Toe and One Leg Standing, Stand To Sit. The physical therapists were assisted by a specific web-based software named CoCo that was developed by Roessingh Research and Development (Enschede, NL; http://www.rrd.nl/en/) in the context of the PERSILLAA project. CoCo is an ICT coaching and counselling system web-accessible to registered users. This application has been developed to suggest to subjects undergoing exercise training the specific physical exercises to be performed in each physical activity session and guide them during their execution. It also helps adjusting the exercise program on the basis of individual and class progress.

### Variables

General health status was measured with EuroQol-5 dimension (EQ-5D), a questionnaire evaluating 5 health dimensions (mobility, self-care, usual activities, pain/discomfort, and anxiety/depression) [34]. At the time of first visit (T0) study participants underwent anthropometric indices assessment (assessment of height, weight, waist circumference, waist-hip ratio, BMI) and their heart rate and blood pressure were also recorded. In order to identify and exclude patients with Alzheimer disease or other forms of dementia, a detailed neuropsychological examination was performed by a registered neurologist also using specific diagnostic questionnaires (QMCI and AD8 tests) [35]. Because study intervention involved a specific physical activity program, the physical activity readiness questionnaire (PAR-Q), which is a widely accepted tool to assess the eligibility to non-competitive fitness programs [36,37], was preliminarily administered to all study participants.

A battery of complementary physical tests from the Fullerton Senior Fitness Test [38] was used to evaluate the physical fitness of study participants [39–42]. More specifically, lower body strength was assessed with the Chair Stand Test (CST) that measures the number of full stands that can be completed in 30 seconds with arms folded across chest; Aerobic endurance was evaluated with the two-minute step Test (TMST) that counts the number of full steps completed in 2 minutes, raising each knee to a point midway the patella and iliac crest. Finally, to evaluate lower body flexibility we performed the Chair sit and reach Test (CSRT) that measures the minimal distance between finger tips and toes when the patient tries to touch his feet from the sitting position [43].

Maximum isometric strength of the hand and forearm muscles was measured with the handgrip strength test using a handgrip dynamometer ARW WAP 80KG [44–46]. The values obtained were considered indicative of sarcopenia if lower than 20 kg for women and 30 kg for men [2].

To assess adherence of the study population to the intervention we took the attendance to each training session and we recorded the data on a centralized web-based database. For each parish we first calculated the number of the attendants to each session and then we added up the number of attendants to the different training sessions to obtain the total number of attendants per parish. Total percent attendance was then calculated for each parish as the percent ratio between the total number of attendants and the product of the number of the study participant of the parish by the number of sessions (which represents the highest possible number of attendants).

### Statistical analysis

Statistical analysis was carried out with IBM SPSS.20 (SPSS Inc, Chicago, IL) and Sigmaplot 11 (Systat, San Jose, CA, USA). Data were examined for normality with the Shapiro-Wilk test and reported as median and IRQ or as mean±SD if normally distributed or not, respectively. Two group comparisons were performed with paired Student’s test or with Wilcoxon paired rank test as appropriate. The threshold for statistical significance was set at p<0.05.

## III. RESULTS

### Study population and adherence to the intervention

A total of 236 older adults expressed their consent to take part to the study and underwent the first visit evaluation. Only 113 of them (median age: 72.0 yr [69.0–75.0]) met the inclusion criteria specified in the methods section and were enrolled in the study. The results of the anthropometric and physical tests that were performed in these subjects and of the self-rated health status questionnaires that they filled out are summarized in Table 1. Overall our study population was composed by young older adults with a high degree of independence, a slight, initial degree of frailty and a normal cognitive status for their age.

Average attendance to gym classes was 40.0 % [30.8–42.0]. 57 of the enrolled subjects showed up at the final anthropometric assessment and 50 underwent the final physical tests. Therefore we could evaluate the efficacy of the intervention only in this group of study completers. The results of this analysis are reported in the next paragraph.

### Effect of the intervention on quality of life, anthropometric parameters and physical fitness

Perceived physical fitness was evaluated by comparing the scores recorded in the *mobility* and *usual activity* fields of the EQ-5D questionnaire. The results obtained showed a significant improvement only in the EQ-5D mobility score with no change in the *usual activity* field. No change was observed in the self-care, pain, anxiety and general health status fields. Total score of the EQ-5D test was significantly higher following treatment suggesting an improvement of the quality of the life of study participants (Table 2).

Significant changes also occurred in anthropometric parameters. We observed a slight increase in BMI that was accompanied by a significant decrease both in waist circumference and in waist to hip ratio (Table 2). Handgrip dynamometer-measured muscle strength significantly increased from 21.1 Kg [16.4–23.5] at baseline up to 24.3 Kg [21.0–28.0] at the end of the intervention (Table 2).

Physical fitness improved as well as indicated by the significant increase in the score of two physical tests measuring physical endurance, the Chair Stand Test and the Two minutes step test (Table 2). The improvement of the results of the *Chair sit and reach Test* showed that the intervention also ameliorated body flexibility (Table 2).

## IV. DISCUSSION

The data reported in the present paper provide the proof of evidence of the efficacy of a physical training program for older people that minimally impacts on their consolidated life habits. This intervention induced a significant improvement in physical performance and enhanced social interactions hence improving the quality of life.

It is well established that older people do not like to break their habits [47–49]. Going to gym, which is an ordinary and simple task for the youngest, may, on the contrary, be a destabilizing, changing-habit event for older people. We reasoned that all together this factor could significantly lower the acceptability of gym programs in older adults. Therefore we designed an innovative approach aiming to embed physical training in their usual day-life activities. We identified the local parish as a potential ecosystem in which our intervention could be introduced with minimal changes in everyday life of study participants. Indeed, our study sample consisted of older women who regularly took part to the activities of their parishes, attending an average of twice/week. Choosing the parish also has the advantage that it is usually close to patient home and that the patient already knows well people attending to its community hence minimizing the detrimental effect of novelty. Obviously, the intervention that we designed was very context-oriented and, therefore, with this study we did not mean to propose a universal model that could be applied in all the social contexts of the Western countries. We intended, instead, to suggest that efforts should be done to identify the microenvironments where ageing people are used to routinely go and where they continue to spend some time together. We used the parishes but in other social contexts, totally different structures, such as, for instance, the Bingo hall could serve the same function.

The efficacy of our intervention in improving the fitness of study participants was documented by the substantial increase in handgrip strength and in physical tests. As expected physical training decreased waist circumference and the waist to hip circumference ratio suggesting that study participants lost fat mass.

In the present study adherence to the gym program was about 40%. Many patients skipped one or more training sessions per month whereas only few of them were fully non compliant, not attending at all to the gym. Although this value of percentage adherence could seem small it is important to emphasize that we obtained it in our population made of older adults that were completely non-compliant to standard physical exercise results, similar to those usually reported in unselected older adults [50].

An important component of our intervention is the use of ICT. Previous investigations clearly demonstrated that ICT may effectively support physical training in older adults. Web-based ICT interventions delivered on different devices such as mobile phones or tables have been shown to increase motivation and help older people correctly performing complex exercises hence enhancing the efficacy of physical training [29,30, 51–54]. An additional feature of the software we used is that it can help adjusting the exercise program to the progress of the class [55]. However, poor computer literacy is still a strong barrier to the implementation of ICT programs in selected social context such as in our study population. Therefore, we decided to include in our intervention also a PC training course as well as a “brain training” app as part of the course. This intervention and its positive effect on cognitive performance will be described in a future paper. It has also to be considered that the improvement in cognitive functions caused by the ICT support could have played a role in the improvement that we observed in physical tests. It has been reported, indeed, that ICT strategies increasing cognitive abilities such as attention and executive functions can help in reducing the impact of physical performance impairment and reduce the risk of falls [56].

A limitation of our intervention is that it was delivered to patients with only a mild impairment of physical performance. All of them were still fully independent and they did not need a continuous support in everyday life. It is unlikely that this model could be successfully extended to patients with a more serious physical impairment because an essential requirement of our intervention is that the patient is still engaged to some extent in social life outside his/her house. It has to be considered, however, that the young older people are the ideal target of any preventive intervention in geriatric medicine because, different from the old-old and the oldest old they have a life expectancy long enough to benefit of the treatment [57].

## V. CONCLUSIONS

In conclusion, we reported preliminary evidence of the efficacy in older women non-compliant to conventional exercise interventions of program that embeds physical training in their every-day life with minimal impact on their habits. Further studies will be required to assess whether adherence can be improved, also in the long term, and to determine the threshold of adherence to obtain an improvement in physical functioning and quality of life.

## 





## Figures and Tables

**Table 1:** BASELINE CHARACTERISTICS OF THE POPULATION STUDY

	n	AVERAGE
Age	113	72.0 [68.0–75.0]
Body weight	113	69.0 [62.8–76.4]
BMI (Kg/m^2^)	113	28.8±4.1
WaistCircumference (cm)	113	92.8±12.1
Hip Circumference (cm)	113	103.0 [100.0–109.6]
Waist to Hip ratio	113	0.88±0.08
Handgrip strength (Kg)	78	20.3 [16.4–23.0]
EQ-D5 (total score)	113	0.73 [0.69–0.85]
Chair Stand Test (number of stands)	113	13.0 [10.0–16.0]
Chair sit and reach Test (cm)	113	6.0 [3.0–8.0]
Two minutes step test (number of cycles)	113	62.5 [40.0–110.0]

Reference values for people in the age range of the study population are as follows: EQ-5D, 0.823 and 0.724 in people aged 65–74 and 75+ years, respectively [34]; Chair Stand Test, < 10 in females aged 65–69 and< 11 if aged 70–74 [58]; Chair sit and reach Test (cm): −1.27–11.43 in females aged 65–69 and −2.54–10.16 if aged 70–74 [37]; Two minutes step test (number of cycles): 75–107 in females aged 65–79 and 68–100 if aged 65–79 [37].

**Table 2:** ANTHROPOMETRIC PARAMETERS AND SCORES AT THE PHYSICAL TESTS AND QUALITY OF LIFE QUESTIONNAIRES IN STUDY PARTICIPANTS WHO UNDERWENT THE FINAL VISIT

	n	BEFORE THE INTERVENTION	AFTER THE INTERVENTION
Body Weight (Kg)	57	70.0 [61.8–75.9]	69.0 [62.8–76.4]
BMI (Kg/m^2^)	57	28.2±3.7	28.8±4.1
Waist Circumference (cm)	57	98.5±10.6	92.8±12.1*
Hip Circumference (cm)	57	106.0 [100.8–111.5]	103.0 [100.0–109.6]
Waist to Hip ratio	57	0.90 [0.88–0.98]	0.88 [0.82–0.93]*
Handgrip strength (Kg)	47	21.1 [16.4–23.5]	24.3 [21.0–28.0]*
EQ-D5 (total score)	62	0.727 [0.689–0.848]	0.796 [0.725–0.849]*
Chair Stand Test (number of stands)	50	12.0 [9.0–15.0]	15.0 [12.0–22.0]*
Chair sit and reach Test (cm between extended fingers and tip of toe)	50	7.0 [4.0–8.0]	0.0 [0.0–5.0]*
Two minutes step test (number of full stepping cycles)	50	55.0 [37.0–100.0]	102.5 [70.0–136.0]*

* p<0.05, paired *t*-test
