# Supplementary material for: An Approach to Prevent Frailty in Community Dwelling Older Adults: a pilot study performed in Campania region in the framework of the PERSSILAA project
Source: Transl Med UniSa. 2019 Jan 6;19:42–8. (PMC6581496)
Supplement: Supplementary file 1 [file TM-19-042-s001.doc]

Table 1. BASELINE CHARACTERISTICS OF THE POPULATION STUDY

|  | **n** | **AVERAGE** |
| --- | --- | --- |
| Age | 113 | 72.0 [68.0-75.0] |
| Body weight | 113 | 69.0 [62.8-76.4] |
| BMI (Kg/m^2^) | 113 | 28.8±4.1 |
| WaistCircumference (cm) | 113 | 92.8±12.1 |
| Hip Circumference (cm) | 113 | 103.0 [100.0-109.6] |
| Waist to Hip ratio | 113 | 0.88±0.08 |
| Handgrip strength (Kg) | 78 | 20.3 [16.4-23.0] |
| EQ-D5 (total score) | 113 | 0.73 [0.69-0.85] |
| Chair Stand Test (number of stands) | 113 | 13.0 [10.0-16.0] |
| Chair sit and reach Test (cm) | 113 | 6.0 [3.0-8.0] |
| Two minutes step test (number of cycles) | 113 | 62.5 [40.0-110.0] |

Reference values for people in the age range of the study population are as follows: EQ-5D, 0.823 and 0.724 in people aged 65–74 and 75+ years, respectively [34]; Chair Stand Test, < 10 in females aged 65-69 and< 11 if aged 70-74 [58]; Chair sit and reach Test (cm): -1.27-11.43 in females aged 65-69 and -2.54-10.16 if aged 70-74 [37]; Two minutes step test (number of cycles): 75-107 in females aged 65-79 and 68-100 if aged 65-79 [37].
