# Supplementary material for: An Approach to Prevent Frailty in Community Dwelling Older Adults: a pilot study performed in Campania region in the framework of the PERSSILAA project
Source: Transl Med UniSa. 2019 Jan 6;19:42–8. (PMC6581496)
Supplement: Supplementary file 2 [file TM-19-042-s002.doc]

**Table 2. ANTHROPOMETRIC PARAMETERS AND SCORES AT THE PHYSICAL TESTS AND**

**QUALITY OF LIFE QUESTIONNAIRES IN STUDY PARTICIPANTS WHO UNDERWENT THE FINAL VISIT**

|  | n | **BEFORE THE INTERVENTION** | **AFTER THE INTERVENTION** |
| --- | --- | --- | --- |
| Body Weight (Kg) | 57 | 70.0 [61.8-75.9] | 69.0 [62.8-76.4] |
| BMI (Kg/m^2^) | 57 | 28.2±3.7 | 28.8±4.1 |
| Waist Circumference (cm) | 57 | 98.5±10.6 | 92.8±12.1* |
| Hip Circumference (cm) | 57 | 106.0 [100.8-111.5] | 103.0 [100.0-109.6] |
| Waist to Hip ratio | 57 | 0.90 [0.88-0.98] | 0.88 [0.82-0.93]* |
| Handgrip strength (Kg) | 47 | 21.1 [16.4-23.5] | 24.3 [21.0-28.0]* |
| EQ-D5 (total score) | 62 | 0.727 [0.689-0.848] | 0.796 [0.725-0.849]* |
| Chair Stand Test (number of stands) | 50 | 12.0 [9.0-15.0] | 15.0 [12.0-22.0]* |
| Chair sit and reach Test (cm between extended fingers and tip of toe) | 50 | 7.0 [4.0-8.0] | 0.0 [0.0-5.0]* |
| Two minutes step test (number of full stepping cycles) | 50 | 55.0 [37.0-100.0] | 102.5 [70.0-136.0]* |

*= p<0.05, paired *t*-test
